# Depressive symptoms and psychosocial stress at work among older employees in three continents

**DOI:** 10.1186/1744-8603-8-27

**Published:** 2012-07-20

**Authors:** Johannes Siegrist, Thorsten Lunau, Morten Wahrendorf, Nico Dragano

**Affiliations:** 1 Department of Medical Sociology, Faculty of Medicine, University of Duesseldorf, P.O. Box 101007, 40225 Duesseldorf, Germany; 2 International Centre for Life Course Studies in Society and Health, Department of Primary Care and Public Health, Imperial College London, London, UK

**Keywords:** Work stress, Depressive symptoms, Older employees, Globalization, Effort-reward imbalance, Job control, Surveys

## Abstract

**Background:**

To assess whether an association of psychosocial stress at work with depressive symptoms among older employees is evident in a set of comparable empirical studies from Europe, North America and Asia.

**Methods:**

Cross-sectional and longitudinal multivariate regression analyses of data from 4 cohort studies with elder workers (2004 and 2006) testing associations of psychosocial stress at work (‘effort-reward imbalance’; ‘low control’) with depressive symptoms.

**Results:**

Cross-sectional analyses from 17 countries with 14.236 participants reveal elevated odds ratios of depressive symptoms among people experiencing high work stress compared to those with low or no work stress. Adjusted odds ratios vary from 1.64 (95% CI 1.02-2.63) in Japan to 1.97 (95% CI 1.75-2.23) in Europe and 2.28 (95% CI 1.59-3.28) in the USA. Odds ratios from additional longitudinal analyses (in 13 countries) controlling for baseline depression are smaller, but remain in part significant.

**Conclusion:**

Findings indicate that psychosocial stress at work might be a relevant risk factor for depressive symptoms among older employees across countries and continents. This observation may call for global policy efforts to improve quality of work in view of a rapidly aging workforce, in particular in times of economic globalization.

## Background

On a global scale, depression is one of the leading causes of premature mortality and disability-adjusted life years [1]. Despite uncertainty about the scope of a potentially increased incidence in recent years [2] depression makes a significant contribution to the global burden of disease and associated costs, in particular in rapidly aging populations [3-5]. On a different level, work stress is now considered a growing threat to the health of employed people, especially so in association with aggravated competition, work intensification and job insecurity resulting from rapid spread of free market principles in a globalized economy [6]. Whether there is an association between work stress and depression has been explored in a number of epidemiological studies (for reviews see [7-10]. Although results are not fully consistent significantly increased odds ratios of incident depression were documented in a majority of cohort studies analysed in these reviews. Given the significance of exposure time for disease incidence one can assume that the association of stressful work with depression is particularly strong among older employees. Yet, this hypothesis has rarely been tested as most studies were conducted in middle-aged working populations [7-10]. It is therefore important to analyse the association of work stress with depression in older employees. A further shortcoming of the current state of the art relates to the fact that most studies testing this hypothesis were restricted to samples of working men and women from a single country or from different countries, mostly within Europe [11-13]. To our knowledge, no study has yet tested the hypothesis in working populations recruited from a variety of countries in different continents.

We set out to fill in these gaps of knowledge by analysing the association of work stress with depressive symptoms in a large sample of older employed men and women (aged 50 to 64) from several countries recruited from Europe, the USA and Asia. We test this association both cross-sectionally (17 countries) and longitudinally (13 countries) (see Methods). Despite their importance in times of growing economic globalization, studies that investigate cross-country comparisons face a considerable methodological problem as it is essential to ensure that the core constructs under study have an identical meaning across different cultures. Moreover, the measurement of these constructs should result in comparable information across the different data sets. In case of clinically relevant depressive symptoms this challenge has been met rather successfully, both at the level of professional judgments based on internationally valid criteria and assessment tools [14-16], and at the level of self-reported standardized questionnaire data assessing depressive symptoms (see Methods).

Can we define stressful work in an identical way and provide a comparable assessment? To answer this question a theoretical model is needed. It is the function of a theoretical model to delineate particular stressful work conditions so that they can be identified at a level of generalization that allows for their use in a wide range of occupations in different socio-economic and socio-cultural contexts. While several such theoretical concepts have been developed [17], two models received special attention in international research and were applied in epidemiological studies in a variety of working populations in different countries, the job strain (or demand-control) model and the effort-reward imbalance model. The job strain model defines work stress in terms of a distinct job task profile where jobs defined by high demands in combination with low decision latitude or low task control are stressful [18]. A complementary model, effort-reward imbalance, focuses on the work contract and the principle of social reciprocity lying at its core. Rewards received in return to efforts spent at work include money, esteem, and career opportunities (promotion, job security). The model asserts that lack of reciprocity (high effort in combination with low reward) occurs frequently in a globalized economy and generates strong negative emotions and psychobiological stress responses with adverse long-term effects on health [19]. Both models are measured by standardized, psychometrically validated questionnaires that are available in a variety of languages [20,21].

In summary, we analyse associations of job strain and effort-reward imbalance at work with depressive symptoms in older employed men and women recruited from countries in three continents, applying validated, cross-culturally comparable methods.

## Methods

### Data

Data were obtained from four longitudinal ageing studies with information from 17 countries: the ‘Survey of Health, Ageing and Retirement in Europe’ (SHARE, Release 2.3.0) [22,23], the ‘English Longitudinal Study of Ageing’ (ELSA, Release 2) [24], the US Health and Retirement Study (HRS 2004 Final V1.0, HRS 2006 Final V2.0, RAND Version J) [25], and the ‘Japanese Study of Aging and Retirement’ (J-STAR) [26]. The four studies were developed in close coordination regarding design and measurements. Countries range from Scandinavia (Denmark and Sweden), England and Ireland, Western Europe (Austria, France, Germany, Switzerland, Belgium, and the Netherlands), Central Europe (Poland, Czech Republic), Southern Europe (Spain, Italy and Greece) to the United States of America (USA) and to Japan. Thus, a variety of countries with different levels of economic development and with considerable socio-cultural diversity are included. Yet, all countries are exposed, to a greater or lesser extent, to the opportunities and constraints of global economic change. All studies except J-STAR are based on representative national samples of individuals aged 50 and older with ongoing waves of data collection in two-year intervals covering a variety of sociological, economic and health-related topics. In J-STAR, a stratified sample from the population of 5 cities was taken which may not reflect a nation-wide representative study group. In all studies, participants were interviewed using Computer Assisted Personal Interviews and self-completion questionnaires. While analyses of cross-sectional associations (covering all 17 countries) use data from the year 2006 only, analyses of longitudinal data include those 13 countries which provide data from two waves (the listed countries without Ireland, Czech Republic, Poland and Japan). Longitudinal associations were explored by linking work stress measures in 2004 with prospective health measured at wave 2 (2006). Since we were interested in work stress in late midlife, samples are restricted to men and women aged 50 - 64 years reporting to do any paid work at assessment of work stress. This restriction resulted in a cross-sectional sample of N = 14,236 persons for the cross-sectional analysis (2006) and of N = 6,657 persons for the longitudinal analysis (2004-2006).

### Measurement

#### Work stress

In all studies work stress was assessed by short versions of validated scales administered by standardized questionnaires. Given the constraints of multi-disciplinary collaborative research within large-scale epidemiological investigations the inclusion of full original questionnaires was not possible. Thus, items were selected on the basis of factor loadings and item-total correlations on respective original scales. With regard to the job strain model, the measurement was restricted to the control dimension [20]. This decision was based on evidence that the predictive power of ‘control’ exceeded the power of ‘demand’ in several studies [24]. Low control at work was measured by the sum score of two Likert-scaled items (1 item in J-STAR), with higher scores indicating lower control at work. For each country, participants scoring in the upper tertile of the score were considered to experience stressful work in terms of low control, in accordance with previously published studies [12,13]. To measure effort-reward imbalance, 2 measuring ‘effort’, and 5 assessing ‘reward’ at work were included (4 items in J-STAR). This selection was based either on the original questionnaire [21] or on its abbreviated, psychometrically validated version [27]. For the selected items, all item-total correlations were far beyond the established threshold of 0.30 [28], ranging from 0.93 to 0.81 (uncorrected) and from 0.67 to 0.42 (corrected). 'Effort-reward imbalance' was defined by a ratio of the sum score of the two scales, adjusted for unequal number of items, where country specific tertiles were calculated [21]. Again, values in the upper tertile were defined as exposure to psychosocial stress at work in terms of this latter model [12,13]. Both questionnaires were included, among others, in large comparative cross-national and cross-cultural studies [29,30], and item response theory was applied to achieve cross-cultural comparability of work stress measurements [29].

#### Depressive symptoms

To measure a clinically relevant indicator of mental health, depressive symptoms were defined on the basis of two internationally established instruments, the Centre for Epidemiologic Studies Depression (CES-D) scale [31], and the EURO-D depression scale [32]. A short validated version of the former scale (8 items) was applied in HRS and ELSA, and J-STAR applied the full version (20 items). In SHARE, the alternative EURO-D (12 items) scale was included. As high degree of comparability of results of the two scales was demonstrated [32,33], respective information was included in comparative analyses. Cut points of the scales indicating clinically relevant depressive symptoms were validated by clinical interviews [34,35]. Several investigations confirmed the cross-cultural comparability of these scales [36-38]. In SHARE, the internal consistency of the EURO-D scale was satisfying with Cronbach’s alpha ranging from 0.62 to 0.78 [37], and similar results were obtained with the CES-D scale [32]. In HRS Cronbach’s alpha of the 8-item version of CESD ranged from 0.81 to 0.83 [39].

#### Additional measures

In addition to gender and age (3 categories), education, employment status and working hours were considered as confounders. For SHARE and ELSA education was measured according to ISCED-97. In the HRS study corresponding levels were obtained based on years of education. Employment status (self-employed versus employed) and a binary indicator of full time work (at least 35 hours per week) were measured. Finally, an additional health indicator, ‘poor self-rated health’, was assessed by a single internationally applied question about one’s self-reported general health, with 5 answer categories ranging from excellent, very good, good, fair to poor, where answers to the last two categories were combined to measure ‘poor health’ [40]. In this analysis we used self-rated health for sensitivity analyses, without presenting detailed results. The frequencies of these variables for the total cross-sectional sample are given in Table 1.

**Table 1:** Distribution of study variables in the 3 world regions*; data from 2006

		**EUROPE (SHARE; ELSA)**		**USA (HRS)**		**Japan (JSTAR)**	
		**Percent**	**N**	**Percent**	**N**	**Percent**	**N**
Gender	Male	52.8	5803	44.7	777	60.1	908
	Female	47.2	5182	55.3	962	40.0	604
Age Group	50-54	39.4	490	28.2	490	26.1	394
	55-59	41.4	701	40.3	701	42.3	640
	60-64	19.2	548	31.5	548	31.6	478
Education	Low	26.6	2845	9.5	164	17.6	266
	Medium	38.6	4130	57.5	995	46.6	702
	High	34.9	3739	33.0	571	35.8	540
Employment status	Self-Employed	18.3	2006	18.7	319	19.6	295
	Employed	81.7	8973	81.3	1390	80.4	1213
Working hours	Part-time	27.4	2961	22.3	378	25.9	376
	Full-time	72.6	7861	77.7	1315	74.1	1075
Effort-reward Imbalance	Yes	31.6	3314	35.0	545	24.8	321
	No	68.4	7167	65.0	1013	75.2	975
Low control	Yes	19.8	2146	25.9	417	11.4	170
	No	80.2	8676	74.1	1195	88.6	1317
Depressive symptoms	Yes	14.1	1536	9.9	170	7.7	100
	No	85.9	9343	90.1	1547	92.3	1202
Self-reported health	Poor health	15.1	1654	13.3	232	9,8	148
	Good health	84.9	9329	86.7	1507	90.2	1359

*17 countries, N = 14, 236 employed men and women.

### Statistical analyses

In a first step, we present the national prevalence of depressive symptoms. Following this, multivariate logistic regression analyses were performed to estimate odds ratios and 95% confidence intervals of depressive symptoms according to the two independently analysed measures of work stress, effort-reward imbalance and low job control. Models were calculated for each work stress scale separately and adjusted for the confounding factors mentioned above. Given the multilevel structure of the European data, we applied multilevel methods where individuals (level 1) are nested within countries (level 2) [41]. By using multilevel modelling, accurate adjustment for country affiliation is possible and the dependence of residuals within a country is considered, since the constant is allowed to vary across countries. These analyses were applied to the full cross-sectional data set and, subsequently, to the restricted data set with two consecutive measurement waves where stressful work, assessed in 2004, was related to depressive symptoms, assessed in 2006.

In the first one of two models, effects were adjusted for additional covariates, whereas a second model they was additionally adjusted for baseline depressive symptoms measured in 2004. By doing so, model 2 estimates how the work stress measures are associated with changes in depressive symptoms between 2004 and 2006 [42]. In keeping with the main focus of this contribution, the globalization of stressful work and its potential impact on mental health, we present the odds ratios separately for the three main world regions from which data are available, Europe, USA, and Asia (Japan). To assess the validity of the analyses sensitivity analyses were conducted. First, analyses were repeated using self-rated health as an additional health indicator. Second, longitudinal analyses were repeated excluding all participants with depressive symptoms at baseline in order to test associations with new onset of depressive symptoms. All calculations were done using STATA 11.

## Results

The prevalence of current depressive symptoms varied considerably between countries, with a majority of countries exhibiting less than 14 percent. Yet, in a few countries a higher prevalence was observed (19,2 percent in Belgium, 23,0 percent in Italy, 26,1 percent in France, and 33,9 percent in Poland). Concerning the three world regions, the prevalence of current depression was lowest in Japan, medium in the USA, and highest in Europe.

To test our hypothesis, multivariate logistic regressions were calculated separately for the three regions of the world. Table 2 presents the results from cross-sectional data. Accordingly, odds ratios of depression were significantly elevated among men and women experiencing work stress in terms of high effort-reward imbalance in all three regions, compared to those who were free from stress at work. The strongest association was observed in the USA, followed by Europe and Japan. A similar trend was obvious for the complementary measure of work stress, low control. However, after adjusting for confounders the association was no longer statistically significant in Japan.

**Table 2:** Associations of work stress with depressive symptoms (multivariate logistic regression analyses) (N = sample with complete data)

		**Depressive Symptoms**		
		**USA**	**EUROPE**	**Japan**
Effort-reward imbalance	Yes	2.28 (1.59 – 3.28) ***	1.97 (1.75 – 2.23) ***	1.64 (1.02 – 2.63) *
	No (Ref.)			
	N=	1510	10047	1083
Low Control	Yes	2.26 (1.57 – 3.25) ***	1.66 (1.45 – 1.90) ***	1.59 (0.88 – 2.86)
	No (Ref.)			
	N=	1560	10342	1226

Odds ratios and 95% confidence intervals (CI). Cross-sectional data from 2006.

Odds Ratios are adjusted for gender, age, education, employment status, working hours.

Given the multilevel structure of the European data, we applied multilevel methods where individuals (level 1) are nested within countries (level 2).

* p < 0.05; ** p < 0.01; *** p < 0.001.

Findings from longitudinal analyses are displayed in Table 3, with the restricted sample of employed and self-employed men and women from 13 countries. Significantly elevated odds ratios of depression, assessed in 2006, are observed among men and women with a high level of work stress in terms of effort-reward imbalance and low control, assessed in 2004. Gender-specific analyses showed no statistically significant differences in effect sizes. Concerning the two statistical models, all odds ratios in the second model are attenuated once baseline depression in 2004 has been taken into account. In this case, elevated odds ratios for both work stress models remain statistically significant in the combined European samples (SHARE and ELSA). Here, an odds ratio of 1.51 (effort-reward imbalance) and of 1.42 (low control) respectively is observed.

**Table 3:** Associations of work stress with prospective depressive symptoms (multivariate logistic regression analyses)

		**Depressive Symptoms (2006)**	
		**USA**	**EUROPE**
Effort-reward imbalance (2004)	Model1	1.94 (1.21 – 3.13) **	1.79 (1.53 – 2.09) ***
	N=	683	6063
	Model2 + symptoms 2004	1.53 (0.91 – 2.57)	1.51 (1.28 – 1.78) ***
	N=	673	6034
Low control (2004)	Model1	1.65 (1.03 – 2.63)*	1.57 (1.34 – 1.84) ***
	N=	714	6246
	Model2 + symptoms 2004	1.46 (0.88 – 2.45)	1.42 (1.20 – 1.68) ***
	N=	704	6213

Odds ratios and 95% confidence intervals (CI). Longitudinal data.

Odds Ratios are adjusted for gender, age, education, employment status, working hours in model 1.

In model 2 outcome depression at follow up is additionally adjusted for depressive symptoms at baseline.

Given the multilevel structure of the European data, we applied multilevel methods where individuals (level 1) are nested within countries (level 2).

* p < 0.05; ** p < 0.01; *** p < 0.001.

The results of additional sensitivity analyses with self-rated health as outcome were similar to those obtained with depressive symptoms. Moreover, sensitivity analyses were conducted to confirm the longitudinal findings. First, we repeated analyses by excluding participants who retired between 2004 and 2006. Second, we restricted our mental health outcome to participants with incident depressive symptoms between 2004 and 2006. In either case, results remained basically unchanged (results not shown). The magnitude and statistical significance of respective odds ratios were well comparable in case of the European and American study samples, both in cross-sectional and longitudinal analyses. However, in Japan, low control was not associated with poor subjective health. Furthermore, instead of analysing the full sample and to adjust for baseline depressive symptoms, we repeated longitudinal analyses by excluding all participants with depressive symptoms at baseline, thus estimating associations with newly manifested depressive symptoms. Again, results were in line with the analyses displayed in Table 3. More specifically, odds ratios in case of effort-reward imbalance were 1.64 (0.87-3.08) for USA and 1.59 (1.29-1.96) for Europe, and in case of low control 1.69 (0.92-3.10) for USA and 1.52 (1.23-1.88) for Europe.

## Discussion

Our results demonstrate that work stress, as measured by core components of two major theoretical models, job strain and effort-reward imbalance, is associated with significantly increased odds ratios of depressive symptoms in cross-sectional and, in part, in longitudinal analyses across different regions of the world. Based on cross-sectional data, the strength of associations is comparable between the three analyses conducted for countries from the three regions Europe, USA and Asia (Japan). Among employed men and women aged 50 to 64 exhibiting a high level of work stress there is an increased probability of depressive symptoms varying from about 60 percent to about 120 percent, compared to those with no or low level of work stress. Findings from longitudinal analyses are restricted to samples from USA and Europe. Although trends are similar to those reported from cross-sectional analyses, effects are attenuated after adjusting for baseline depression, where elevated odds ratios remain significant in Europe only – a finding that might be explained by the large sample size compared to the US sample.

Taken together, our results lend preliminary support to the notion that work stress is associated with an elevated probability of experiencing clinically relevant depressive symptoms in employed men and women of early old age recruited from a variety of countries in different regions of the world. Observed effects remain statistically significant after adjustment for gender, age, education, employment status, working hours and country, but adjustment for baseline depression systematically reduces the size of effects in longitudinal analyses. This latter observation is well known from other investigations and may in part be due to common method variance between two sets of simultaneously assessed self-report measures [43]. Our findings are consistent with those reported from a single country or from studies comparing European countries [11-13,44].

To the best of our knowledge, this is the first report demonstrating an association of work stress with depression in early old age populations across a variety of countries from different regions of the world, using identical or highly comparable measurements of the constructs under study. However, there are several limitations to our analysis. First, the number of countries from which longitudinal data are available is smaller than is the case with cross-sectional data. Therefore, respective findings cannot be generalized to all samples under study. Moreover, results are restricted to the specific age group, and the Japanese sample was not intended to be representative of the respective national population group. Inclusion of more Asian countries would provide more robust estimates. In fact, two more aging studies with comparable study designs have been initiated meanwhile, in South Korea and in China, thus offering an extension of the current analysis in future research.

Second, we restricted the analysis of work stress to two core theoretically grounded notions of stress, lack of control and failed reciprocity between effort and reward [45]. Although these theoretical notions are particularly relevant in delineating stressful working and employment conditions in the context of economic globalization future studies may broaden the range of work-related stressors, e.g. by including organisational injustice [8] and precarious work [46]. However, given the restrictions of applying short questionnaires in multi-disciplinary international investigations we were not in a position to measure all components of the two models with complete scales and to include additional theoretical notions. Yet, rather than overestimating the effects of work-related stress, this may have led to underestimating effect sizes.

A third limitation concerns the measurement of depression. Despite satisfactory criterion validity the two self-administered scales measuring depressive symptoms may not capture clinically relevant conditions to a sufficient extent, as is the case with an established standardized approach based on clinical interviews [15]. Yet, correlations of the two scales, the CES-D scale and the Euro-D scale, were high, and both scales demonstrate convincing psychometric properties. Fourth, cultural variations in experiencing and expressing stressful work as well as depressive symptoms may not have been adequately captured by short standardized questionnaire methods [15]. Therefore, in addition to applying item response theory to improve cross-cultural comparability of the scales [29] qualitative studies are desirable to validate these preliminary findings [47]. A more comprehensive understanding is expected from such studies, specifically in case of explaining the observed differences in effect sizes of work stress on depression between Japan and the other countries or between men and women (although, in our data, gender differences were not significant). In this context, socio-cultural differences in recall of depressive symptoms may also need a more detailed analysis [48]. Finally, information on mental health in this analysis was restricted to depressive symptoms. The inclusion of additional health indicators would further support the hypothesis. In fact, in sensitivity analyses we repeated all calculations presented above with data on ‘poor self reported health’ as an outcome criterion. Results were highly comparable to the ones given in Tables 2 and 3. In further sensitivity analyses excluding participants who retired between 2004 and 2006 and focusing on incident depressive symptoms findings were almost identical to the ones presented in the Results section (results not shown).

These limitations are balanced by several strengths. All epidemiological studies on aging populations were designed in close coordination, and the data collection procedure met internationally established quality standards [22-24]. The questionnaires applied to measure the core constructs, work stress and depression, were either fully identical or well comparable, thus offering opportunities to conduct intercultural comparisons. Furthermore, the four cohorts represent a relatively homogenous age group, 50 to 64 years, where exposure to work stress can be assumed to have occurred for a couple of years or even decades. In this age group, the prevalence of depressive symptoms is substantial, and the policy dimension of this fact is indicated, among others, in the prominent role of depression as one of the most frequent diagnoses justifying disability pensions in several countries included in this study [3]. Finally, the two work stress models underlying this analysis were successfully applied to explain stress-related mental and physical disorders in a large number of prospective epidemiological studies [10,12,44,49], and the psychobiological pathways linking lack of control and frustration of rewards to disease were tested in a variety of experimental studies [50,51].

What are the policy implications of these results? At the international level, coordinated efforts are needed to strengthen measures that mitigate health-adverse effects of stressful work, e.g. by reducing the impact of neoliberal policies on labour market standards and quality of work. At the national and local levels, investments into worksite health promotion programmes and occupational health services targeting occupational high risk groups contribute to a reduction of the burden of stress-related disorders, given the high prevalence of low control and effort-reward imbalance amongst the workforce with low qualification [52]. Preliminary favourable results from intervention studies that increase control and reward at work by implementing theory-based organizational changes support this notion [53].

## Conclusion

In conclusion, our results lend preliminary support to the notion that work stress is associated with an elevated probability of experiencing clinically relevant depressive symptoms in early old age working men and women recruited from a variety of countries in different regions of the world. If supported by further evidence these findings call for preventive efforts that aim at improving the quality of work and employment at national as well as international levels. Such efforts are needed in view of the powerful and partly health-adverse effects of work and employment in times of economic globalization.

## Competing interests

The authors declare that they have no competing interests.

## Authors’ contributions

All authors read and approved the final manuscript. JS proposed the analysis and drafted the manuscript. TL and MW jointly conducted the empirical data analysis, helped with literature search and revised the draft. ND contributed to the study design, revised the manuscript and contributed to its final version.
